# Six ‘biases’ against patients and carers in evidence-based medicine

**DOI:** 10.1186/s12916-015-0437-x

**Published:** 2015-09-01

**Authors:** Trisha Greenhalgh, Rosamund Snow, Sara Ryan, Sian Rees, Helen Salisbury

**Affiliations:** Nuffield Department of Primary Care Health Sciences, University of Oxford, New Radcliffe House, Radcliffe Observatory Quarter, Woodstock Road, Oxford, OX2 6GG UK

## Abstract

**Background:**

Evidence-based medicine (EBM) is maturing from its early focus on epidemiology to embrace a wider range of disciplines and methodologies. At the heart of EBM is the patient, whose informed choices have long been recognised as paramount. However, good evidence-based care is more than choices.

**Discussion:**

We discuss six potential ‘biases’ in EBM that may inadvertently devalue the patient and carer agenda: limited patient input to research design, low status given to experience in the hierarchy of evidence, a tendency to conflate patient-centred consulting with use of decision tools; insufficient attention to power imbalances that suppress the patient’s voice, over-emphasis on the clinical consultation, and focus on people who seek and obtain care (rather than the hidden denominator of those that do not seek or cannot access care).

**Summary:**

To reduce these ‘biases’, EBM should embrace patient involvement in research, make more systematic use of individual (‘personally significant’) evidence, take a more interdisciplinary and humanistic view of consultations, address unequal power dynamics in healthcare encounters, support patient communities, and address the inverse care law.

## Background

All authors have research experience and academic qualifications, but we are patients and carers as well (see ‘Details of Contributors’ below). Some of us were patients and carers first, then became academics; some were established academics before illness led us to reframe our perspective on evidence-based medicine (EBM).

Incorporating the patient’s perspective in EBM is sometimes conflated with ascertaining his or her preferences and sharing decisions about possible tests and treatments. These are important elements of good practice (covered in separate papers in this series [1, 2]), but they comprise a small fraction of what healthcare is [3, 4]. Furthermore, whilst we applaud the EBM community’s rapidly emerging interest in the patient perspective, we are concerned that a narrow, doctor-defined ‘patient’s agenda’ – epidemiologically-based and focused on a set of choices to be made during the medical encounter – is being imposed, with the best of intentions, on people who live with illness.

An ‘evidence-based’ healthcare decision is inevitably informed by the stages of evidence creation. First, some people – traditionally researchers and/or doctors, but increasingly with patient and carer input – decide which outcomes count. Next, research is undertaken to find out how to best achieve the designated outcomes. Results are published and, later on, a clinician interprets and shares them in the clinical encounter.

The patient in the above scenario begins from a different place. Even when patients are ‘informed’, ‘empowered’, and ‘health-literate’ (and especially when they are not), they rarely inhabit a world of controlled experiments, abstracted variables, objective measurement of pre-defined outcomes, average results, or generalizable truths. Rather, they live in the messy, idiosyncratic, and unpredictable world of a particular person in a particular family context (or, for some, in a context of social isolation and/or abandonment by family) [5, 6]. Notwithstanding this, patients may seek out medical information and self-monitor biometric variables, with or without the knowledge or support of their clinician [7]. A patient’s symptoms and measurements, along with the implications, factors at stake, and potential trade-offs of different management options, are likely to be discussed with family, friends, and peers [8]. The clinical encounter, whether patient-initiated (e.g. to present a symptom or concern) or clinician-initiated (e.g. an invitation for screening or chronic disease surveillance), has cultural and moral significance and occurs against a complex backdrop of personal sense making, information seeking, and lay consultations [9–11].

The options tabled by the clinician for a ‘shared decision’ may or may not resonate with what has occurred in the patient’s world up to this point. Furthermore, following a (more or less) shared decision, the patient goes away and re-enters what has been termed the ‘lifeworld’ [12] – a world where people rather than biomedical variables have salience and where it is particularities, not mean values or generalizable truths, that matter [13]. In this world, different factors will be at stake; the illness as lived will differ from the disease or risk state in the evidence-based guideline, and may well be at odds with the outcomes (whether ‘patient reported’ or not) measured in the research trial [14]. With the help of particular carers, family, friends, and peers (whether defined as ‘carers’ or not), the patient tries to align the evidence-based model of disease with the actual experience of illness or (assigned) risk.

Below, we discuss six features of EBM – which we refer to, figuratively, as ‘biases’ – that may inadvertently devalue this broader patient and carer agenda: (1) the lack of patient input to the research process; (2) the low status given to experience (‘anecdote’) in the hierarchy of evidence; (3) EBM’s tendency to conflate patient-centred care with use of shared decision-making tools; (4) the limited attention given in EBM to power imbalances that suppress the patient’s voice; (5) EBM’s over-emphasis on the clinician-patient dyad (overlooking the ongoing work of self-management and the importance of the patient’s wider social networks, both on and offline); and (6) EBM’s primary focus on people who seek and obtain care (rather than on the hidden denominator of those that do not seek or cannot access care). These influences, and their potential effects on the process and outcome of evidence-based care, are summarised in Table 1. We consider them in turn below.

**Table 1:** ‘Biases’ against patients and carers in traditional evidence-based medicine (EBM) and how they might be overcome

Nature of bias	Impact on process of care	Impact on outcome	How this bias might be minimised
1. Most published research had minimal patient input	Example: evidence relates to options and outcome measures that patients themselves would not have chosen	The available menu of evidence-based choices reflects a biomedical framing and omits options that might be more acceptable and effective	Patient and public input to setting research priorities, study design, choice of outcome measures, and interpretation and dissemination of findings must be prioritised and effectively resourced
	Recruitment methods to trials address only a fraction of the population	Study findings apply only to this sub-population	Diverse and questioning patient/carer steering group may help recruit more diverse and representative samples
2. EBM’s hierarchy of evidence tends to devalue the patient or carer experience	Abstracted evidence from population samples is given more weight than real, individual evidence from this patient/carer	The patient is effectively ‘regressed to the mean’ and offered the option(s) that the average patient would benefit most from	‘Personally significant evidence’ from a particular patient in the here and now should be systematically captured and treated as complementary to ‘statistically significant evidence’ from distant research populations
	Qualitative evidence, even when robust and relevant, is rarely used to its full potential	Personalisation of care lacks nuance and context, because research addressing ‘how’, ‘why’, and ‘in what circumstances’ has not been used	Narrative, phenomenological, and ethnographic research designs should be viewed as complementary rather than inferior to epidemiological evidence – though qualitative, like quantitative, research must be appraised for rigour and relevance
3. EBM conflates patient-centredness with use of shared decision-making tools	The ‘patient’s agenda’ is framed through a medical lens and reduced to a series of decisions about tests and treatments	Humanistic aspects of the consultation (empathy, compassion, the therapeutic alliance) are devalued and may be overlooked	Working with humanities scholars and psychologists, EBM researchers should acknowledge and incorporate interdisciplinary approaches to extend and complement their current focus on shared decision-making
4. Power imbalances may suppress the patient’s voice	Much of the patient’s agenda will not get aired in the consultation	Advice that is given, and management plans that are ‘agreed’, may be ignored (but may be inappropriate anyway since they are based on a partial picture)	Working with social and political scientists, EBM researchers should collect and apply evidence on how to make consultations more democratic (see main text for examples)
5. EBM over-emphasises the clinical consultation	Clinicians underestimate the extent of self-management and the value of lay networks (in which people support and inform one another) both face-to-face and virtual	Clinicians and researchers focus on ‘interventions’ that they can deliver instead of considering how they can support models of care in which they are no longer central	Working with social scientists, EBM researchers should become comfortable with naturalistic designs for studying the patient in a real-world context and exploring the dynamics of social networks and online groups from a complex systems perspective
6. EBM is concerned mainly with people who seek (and can access) care	People with greatest need for evidence-based care are least likely to receive it	A ‘hidden denominator’ of hardest-to-reach sub-populations may remain undiscovered, hence EBM may appear to have solved more problems than it actually has	EBM researchers should embrace more explicitly a public health agenda, in which preferred study designs may be observational and developmental (including participatory co-design) rather than controlled experiments

Note that whilst all the ‘biases’ below are evident in the EBM literature, we are not suggesting that practitioners, researchers, or teachers of EBM are, as individuals, biased (that is, prejudiced) against patients or carers. On the contrary, many protagonists of EBM are passionately committed to working in a patient-centred way. Our argument is that despite the best intentions of these individuals, EBM’s paradigmatic assumptions, theories, tools, and techniques, as well as its existing evidence base, contain potential distortions that may have negative consequences for the people it aims to serve. In short, it is the paradigm that contains the biases highlighted below, not (in general) the people who seek to develop or apply it. We hope that practitioners, teachers, and researchers of EBM will ask themselves when reading each of the biases below: “Given that I personally seek to be unbiased in relation to patients and carers, how should I alter my use of evidence/teaching approach/research focus to help redress this bias?”

## Discussion

### Bias 1: Most published research has had minimal patient input

Evidence generated by clinical research will depend on who asks the questions, who defines the outcome measures, who interprets the findings, and who disseminates the outputs. In the past few years, many research funders have encouraged patient input to each of these steps [15]. However, it will be decades before this laudable stance achieves the necessary change in the knowledge base so that it truly reflects patients’ priorities and needs. Most studies underpinning today’s evidence-based decisions were designed in an era when researchers were assumed to know better than patients which interventions should be compared, which outcomes should be measured (and when), what the data meant, and who should be informed of the results.

In the widely cited Diabetes Control and Complications Trial (DCCT), for example, conducted between 1983 and 1993, people with type 1 diabetes were randomised to ‘intensive’ or ‘conventional’ treatment and followed-up long term to assess the risk of complications [16]. Whilst intensive treatment was associated with a lower incidence of microvascular complications (including the presence of asymptomatic microalbuminuria, a surrogate endpoint that clearly mattered to the researchers), it tripled the incidence of severe hypoglycaemia – a complication classified as ‘minor’ by the researchers since it was not, on average, associated with cognitive decline or lower quality of life. Indeed, the only kind of hypoglycaemic attack counted as a problem in the DCCT was one “*in which* [medical] *assistance was required in the provision of treatment*” [16].

When the DCCT was set up, people with diabetes were not invited to help design or oversee it (reflecting prevailing research practice at the time). Those who have experienced hypoglycaemic episodes may have different views on how necessary it is to avoid such experiences. One problem with frequent hypoglycaemic episodes is (possibly permanent) loss of awareness of impending hypoglycaemia – a phenomenon that people with type 1 diabetes consider important and dangerous [17]. The DCCT researchers’ conclusion – that a policy of tight diabetes control should be routinely pursued – was based largely on their own value-judgement that delay of microvascular complications was worth the trade-off of a substantial increase in the incidence of hypoglycaemic attacks severe enough to impair consciousness. After cataloguing the comas, seizures, and fatal motor accidents caused by hypoglycaemia in the study, they concluded: “*Although we are mindful of the potential for severe injury, we believe that the risk of severe hypoglycaemia… is greatly outweighed by the reduction in microvascular and neurologic complications*” ([16], p. 983).

A similar conclusion was drawn about tight control of type 2 diabetes based on the UK Prospective Diabetes Study, conducted between 1977 and 1997 [18]. Participants, newly diagnosed with type 2 diabetes, were randomised to tight or conventional glycaemic control. Tight control (achieved with insulin or oral medication) was associated with a 12 % lower risk of what the trial authors called “*any diabetes-related endpoint*” (that is, clinical end-points predefined by the researchers) and a 25 % lower risk of microvascular complications (including microalbuminuria). Tight control with insulin was associated with a significant increase in both weight gain and hypoglycaemic episodes. Again, patients were not formally consulted either when designing the trial or when interpreting findings. The study’s authors and journal editors interpreted the findings to support a policy of tight glycaemic control in type 2 diabetes [19].

The questionable evidence from DCCT and UK Prospective Diabetes Study directly informed the UK Quality and Outcomes Framework, a pay-for-performance scheme in which general practitioners were financially incentivised to monitor and manage diabetes and other conditions in a stipulated way [20]. The Quality and Outcomes Framework target introduced in 2008 (an HbA1c of below 7.0 %) reflected what policymakers deemed the evidence base for tight glycaemic control from these early trials (and which others have dubbed ‘the idolatry of the surrogate’ [21]). It ignored more recent evidence from the larger ACCORD trial, which showed little (if any) benefit from tight versus conventional control and an increase in mortality with the former [22, 23]. As a result, many people with diabetes were treated aggressively by doctors whose personal income depended on achieving outdated and dangerous biomarker target levels [24], increasing the risk of recurrent hypoglycaemia and its associated hard-to-capture impacts on quality of life. Whilst the target was revised a few years later to 7.5 % by the National Institute for Health and Clinical Excellence [25], it is possible (though by no means certain) that attention to patient priorities at the time the DCCT and UK Prospective Diabetes Study trials were designed, executed, and interpreted might have prevented this potentially harmful policy being introduced.

The transition from ‘outcomes that matter to researchers’ to ‘outcomes that matter to patients’ has fuelled (and been fuelled by) the rapidly expanding science of patient-reported outcome measures – standardised instruments developed via systematic surveys of people who have the condition being researched [26]. Factoring in the patient perspective in trial design is an important step forward. Nevertheless, patient-reported outcome measures and similar instruments – which effectively give us patients’ priorities ‘on average’ – can never fully capture the situated, fluctuating granularity of what matters most to a particular patient and carer at a particular point in the illness journey (including why the person has or has not consulted the clinician at a key decision point). We consider this agenda in the next few sections.

### Bias 2: EBM’s hierarchy of evidence devalues the individual patient experience

Standardised measures of patient priorities are less relevant when dealing with individuals. If we want to tailor an evidence-based decision to a particular patient’s priorities and circumstances, we need data that are personally significant in the here and now – and for this we need the richness of narrative.

The individual case report sits at the bottom of EBM’s hierarchy of evidence. Indeed, we are explicitly warned not to trust ‘anecdotal’ evidence [27]. This is entirely appropriate if the question being asked is “should I rely on a story of what happened to some other patient when advising this patient?” However, the warning is misplaced – harmful even – if the question is “what do I know about this patient that will help me work with him or her to refine and personalise a management plan?” The latter question demands that statistically significant evidence from research trials is interpreted and applied with an understanding of the personally significant evidence of the patient’s own experience. Personally significant evidence includes both objective evidence (e.g. what this patient’s test results show), and subjective evidence (e.g. what this patient feels; what matters to him or her) [28].

For example, if I have taken my daily statin on thousands of occasions without developing muscle pains, and if my blood tests show no rise in marker enzyme levels, the chance that I will develop muscle pains on the same statin tomorrow is much less than the published incidence of myalgia on this drug, based on the mean incidence measured in thousands of patients in post-marketing surveillance studies. Clearly, judgement is needed when deciding how much weight to give personally significant evidence compared to statistically significant evidence derived from a distant population sample.

EBM is defined in the literature as the science of integrating the clinician’s expertise and judgement with best research evidence and the individual detail of the patient’s case [29]. It emerged partly as a reaction to widespread inconsistencies in clinical decision-making (such as managing one patient on the basis of what happened to the previous patient) – and has been very successful in improving outcomes. However, whilst the science of ‘best research evidence’ has moved on substantially, the EBM literature has paid much less attention to the science (and art) of how to capture the subjectivity, uniqueness, and real-world messiness of the individual case and how to integrate it with research data to aid decision-making. Similarly, many people in the EBM community acknowledge that qualitative research to describe the patient experience, including the perspective of carers and significant others, can add granularity and meaning to research findings consisting of effect sizes, confidence intervals, and grand means. Nevertheless, they also tend to retain a hierarchical view of the value of such research, viewing qualitative evidence as less robust than quantitative evidence, rather than complementary to it and addressing different questions.

Not all individual patient experiences are research data, of course [30]. However, systematically collected narratives, along with phenomenological and ethnographic evidence (studies of the lived experience of illness and healthcare), provide essential counterweight to the epidemiologically oriented framings and categorisations of EBM. Findings from such research include that:

- The EBM literature tends to depict the patient’s illness as a fixed entity with more or less stable properties; it often portrays the patient as feeling the same about their condition tomorrow as they do today. In reality, symptoms of chronic illness can fluctuate substantially from day to day, as does the significance a person places on the illness [31–33].
- Being ill is a tiny part of what it means to live with a long-term condition (especially one that is largely asymptomatic). Most of the time, it is the living that is foregrounded, not the illness [29]. The EBM literature tends to depict a long-term condition as deviation from an assigned ‘normal state’ (measured by biomarkers) and as periodic ‘illness exacerbations’ that prompt the patient to seek care. The patient’s experience of the same condition may not be as an illness at all but as a dimension of being, a fact of life, and something that must be attended to and ‘tinkered with’ [31, 34, 35].
- Much of the EBM literature relies on (and its practitioners must to some extent accept) fixed categories and definitions of what a disease is. Qualitative research can inform new categories and definitions if researchers are open to this possibility. Patients with depression, for example, who took selective serotonin reuptake inhibitors, were ignored for years after they raised concerns about side effects such as ‘electric head feeling’ that did not fit the existing ‘evidence-based’ model of the drug’s effects or the formal categories of adverse events used in standardised post-marketing surveillance [36].

### Bias 3: EBM conflates patient-centredness with use of shared decision-making tools

Few people think of their illness(es) as a series of discrete decision nodes. Being presented with a menu of options, each tagged with a probability, odds ratio, number needed to treat, or number needed to harm (even when the last two are expressed visually as so many happy or sad faces, respectively) can be problematic, even for those who do. Option grids and other ‘tools to support conversations’ represent significant progress in the shared decision-making field, but remain little used [1, 37].

One reason for the limited success of decision aids is that the patient is not a dispassionate information processer. In contrast to the autonomous rational chooser assumed in EBM’s decision trees, we make many of our life choices for reasons other than effectiveness or efficiency – for example, because we think a particular option would fit in with family plans, align with cultural expectations of good parenting, or honour the memory of an ancestor [3–5]. Unless these reasons are recognised as primary drivers of human behaviour, clinician and patient will be at cross-purposes.

Communication is only partly about sharing information and agreeing a management plan; it also involves talk and gestures to establish and strengthen a therapeutic relationship [38]. The therapeutic relationship is central, not marginal, to evidence-based practice. The stronger it is, the greater the chance that there will be a mutually agreed management plan, the more comfortable the patient will be carrying out their part in the plan and the more satisfied both parties will be [39, 40].

There is strong and consistent evidence that the success of the evidence-based consultation depends on its humanistic elements as much as on what information is shared and how. It is nearly 30 years since family medicine introduced the ‘patient centred clinical method’ [41, 42], summarized in a recent review as: “*the adoption of a biopsychosocial* [incorporating EBM, psychology and attention to social context] *perspective by providers; the sharing of decisions and responsibilities between patients and providers; the strengthening of practitioners’ compassion, sensitivity to patients’ distress and commitment to respond to patients with empathy in an effort to alleviate suffering*.” [43].

As Miles and Mezzich have observed [44], there is remarkably little overlap between the EBM movement (oriented to objective, scientific, and often mathematical management of disease and risk) and the movement for patient-centred care (“*the … imperative to care, comfort and console as well as to ameliorate, attenuate and cure*”). The time is well overdue for these two important streams of scholarship in clinical method to explore their differences and establish common ground.

### Bias 4: Power imbalances can suppress the patient’s voice

Healthcare interactions are characterised by socially prescribed roles and by imbalances of power and status that profoundly affect how each party behaves [9]. In the medical consultation, for example, the doctor has higher status, greater familiarity with the system, (usually) greater knowledge of disease process, and more extensive access to further information and resources. The doctor also typically controls the agenda and the use of time; he or she selects the language used to define and record the problem (and decides whether the patient’s account is sufficiently important and credible to be worth recording at all). The doctor can ask the patient to remove clothing and reveal intimate and embarrassing aspects of their body or mind, and ask a valued carer to leave the room for reasons of ‘confidentiality’.

Whilst a clinician’s use of power may be appropriate and inevitable (to the extent, for example, that when we are sick, our capacity is impaired and we want to be looked after and for highly-trained professionals to make decisions on our behalf [45]), they can sometimes distort interaction in a way that disadvantages the patient – especially when the doctor is under time pressure and/or not behaving altruistically [46], when doctor and patient are from different social classes or speak different languages [47], or when the patient’s complaint fits poorly with the biomedical model of disease [48, 49]. There may be no truly democratising solution to this ‘bias’, since illness makes us vulnerable, doctors are (at least in theory) experts on the condition being treated, and the goal of equal power-sharing may turn out to be (as one reviewer of an earlier draft of this paper put it) a “*race to the bottom*”.

However, even when patients have greater knowledge about their condition than the doctor treating them, the power dynamic is such that the doctor’s (in this example, weaker) evidence tends to trump the patient’s (in this example, stronger) evidence – and the former may succeed in defining the latter as ‘non-compliant’ [10, 50]. In one qualitative study of people with type 1 diabetes, although specialist doctors supported “*participatory decision making*” and empowerment of patients, they frequently discounted patients’ experiential knowledge and withheld resources that would allow patients to make truly informed decisions [51].

Examples from these studies included doctors dismissing symptoms that were not explained by blood tests, ignoring patient experience that did not correspond to textbook descriptions, using medical jargon to re-establish a position of power, and actively withholding information or services. Patients learnt to conceal their own expertise and treatment decisions in order to comply with medical expectations and to avoid professionals becoming “*patronizing or angry*” [50, 51]. All these might be considered as examples of what has been called ‘epistemic injustice’ – that is, the numerous and often subtle ways in which patients may be dismissed in their specific capacity as knowers [52].

Power imbalances between clinicians and patients are particularly stark in the mental health field, where the doctor has the power (in consultation with other professionals) to declare the patient as ‘lacking mental capacity’, incarcerate him or her, and impose treatment. The mental health literature contains troubling examples of people who consider themselves to have been dehumanised in the name of evidence-based practice and who now describe themselves as a ‘survivor movement’ (that is, those who have survived medical interventions that did them alleged harm) [53, 54].

For all these reasons, those who seek to make consultations ‘evidence based’ need to pay more nuanced attention to the power dynamics in these interactions. Measures, such as allocating more time to the consultation, using advocates and mediators, encouraging patients to bring lists of concerns, explicitly recognising and addressing the differing needs of disadvantaged groups and visiting vulnerable patients in their homes, and encouraging patients to bring a carer or advocate into the consultation if they wish, for example, are all evidence-based ways to reduce the power imbalance in the patient’s favour [55–61].

A reviewer of a previous draft of this paper pointed out that the power imbalances described in this section may also play out when patients and carers are invited to be involved in research. Offering lay people the opportunity to help design studies and challenge researchers’ assumptions and perspectives may not always translate into democratic partnerships, especially in situations where power-knowledge imbalances are prominent.

### Bias 5: EBM over-emphasises the clinical consultation

Shared decision-making is strongly emphasised in EBM, but this focus assumes that the key interactions occur between a patient and a clinician around a medical decision tree. This depiction is flawed on a number of fronts.

First, we are highly social and mutually dependent beings. Our interactions with medicine often involve others (who may be present or absent during the consultation) [62, 63]. Managing a chronic illness involves work, which is typically distributed across a network of family and friends [3, 8, 31, 64–66]. Doctors generally know this, but their ‘evidence-based’ discussions with patients about the options for tests and treatments rarely take full account of which people and perspectives the patient would like to bring into the conversation, when, and how; this is of more than tangential significance. Older couples, for example, may be managing various conditions and other life problems concurrently, and may develop a hierarchy of priority. In such circumstances, ‘being ill’ becomes a negotiated position depending on one’s responsibilities and commitments to others [3, 64].

Second, the overwhelming majority of decisions about a person’s chronic condition are made by that individual, their carer(s), and their lay networks without the input of professionals [10, 67]. The knowledge of how to manage one’s own illness overlaps only partially with the knowledge that doctors draw on to manage diseases; it also includes the embodied, tacit knowledge of particular symptoms and the body’s response to treatment [3, 68]. Some decisions (such as which drug to take, if any) may be best shared with one’s clinician; others (such as how to tell one’s employer about illness or how to cope emotionally with stigma) may be better shared with friends or fellow patients. Tacit knowledge is the stuff of communities of practice – accumulated through years of experience and exchanged through stories [69–71]. A particularly revealing genre of patient narrative is doctors’ stories of their own illness journeys – in which they reveal how little they knew about their condition before experiencing it themselves, and how much they learned, often slowly and tangentially, from hearing or reading stories from other patients [72–75].

Mutual support and knowledge exchange among people with long-term conditions is not a new phenomenon, but its form is changing. Old-style patient support groups that met periodically in a local venue, perhaps supported by national or local charities [76, 77], have been joined by virtual peer support groups (e.g. on Facebook, Twitter, or bespoke online communities that may be supported by the healthcare service provider) [78, 79]. Members value knowledge exchange (both explicit and tacit) as well as practical tips and emotional support [80–83].

Tacit knowledge (personally embodied, socially shared) is captured poorly if at all in the design of the clinical trials underpinning EBM, which focus predominantly on discrete ‘interventions’ that doctors and other health professionals can offer their patients (drugs, operations, specialist technologies, education). Herein lies a paradox: clinician-researchers are building an experimental science of how they can intervene in patients’ illnesses [84], while patients themselves are building collaborative communities aimed at supporting and informing one another [80–83]. Hence, EBM’s accumulating body of (explicit, research-based) knowledge and the (informal, tacit, and socially shared) knowledge actually being used by people managing their condition are developing separately rather than in dialogue with one another.

Lay networks and online support groups emerge and change organically. They are complex systems that cannot be experimented on or ‘controlled for’ [85, 86]. They exchange the kind of knowledge that is (by definition) hard to define or quantify. As such, they cannot be understood purely through the kind of research designs with which the majority of the EBM community is familiar. Yet, if it is to remain relevant, EBM must engage with these communities and, to do so, EBM scholars must learn a new language and methodology – that of the social science of networks and digital communities [87, 88].

### Bias 6: EBM is concerned mainly with people who seek care

The EBM process is classically depicted as starting when the patient presents to the health service and the practitioner is encouraged to ‘ask a focused question’. The reality for many sick or at-risk individuals is that getting to see a health professional is a significant hurdle – or else an option that, for whatever reason, they have not yet come to contemplate. As a result of this ‘hidden denominator’ of people who do not seek or cannot access care, clinic populations will be unrepresentative and findings from research on these populations will be systematically biased.

As Hart argued decades ago in his paper ‘*The Inverse Care Law*’, because of the distorting and mutually reinforcing impact of the social determinants of health (such as poverty, low health literacy, social exclusion, and so on), and the limiting impact of illness itself on people’s physical and mental capacity, individuals most in need of healthcare are least likely to seek it or receive it [89].

It is no coincidence, for example, that the recent Confidential Inquiry into Premature Deaths of People with Learning Difficulties in the UK attributed many such deaths to complex interactions between physical, cognitive, and social factors, including, in many cases, not being able to access the care needed to prevent an otherwise avoidable death [65]. Through ignorance, stereotyping, or cognitive biases, doctors may fail to recognise general medical or surgical conditions in someone who is known to the system as a ‘mental health’ or ‘learning disabled’ patient [90–92]. A recent BMJ series has highlighted the crucial importance of ‘mundane’ design features of hospitals such as car parking and the helpfulness of booking clerks on their accessibility to disempowered patients [93].

Making sense of the inverse care law is complicated and requires us to develop and test theories as well as simply measuring variables. For example, Dixon-Woods et al. [94] undertook a systematic review of the qualitative literature on barriers to access. Using sociological concepts, they developed the notion of candidacy – the way in which health services define (and continually redefine) who is ‘eligible for’ and ‘deserving of’ particular tests and treatments, and in which people in turn come to define what counts as an illness needing care. These processes are dynamic and mutually shaping – and they profoundly influence who ends up in the denominator population against which the real-world effectiveness of tests and treatments gets assessed. A good example of candidacy is how learning-disabled individuals may have to fight for the ‘right’ to be resuscitated [95].

Andersen and Vedsted used ethnography to document the ‘logic of efficiency’ that pervaded a Swedish healthcare organisation [96]. They showed how patients, in order to gain access to its services, had to ‘juggle’ this logic of efficiency (that is, continually reframe their symptoms and concerns to fit organisational categories) in order to deal with uncertainties and complex needs – and some were more adept at this than others.

EBM’s tendency to focus on the clinical encounter (rather than the wider context in which people get ill or the cultural logics that shape organisational systems) means that long-term conditions are assessed and treated primarily in terms of individual risk factors and behaviour choices. However, ‘individual behaviour choices’ is only one way of framing this issue. Another approach, preferred by public health practitioners, is to consider how the wider environment shapes and constrains the behaviour of individuals (whose de facto choices may therefore be limited) and introduce system-level changes that make particular choices easier to make.

The built environment in any locality, for example, can be more or less obesogenic, unsafe, dementia-unfriendly, and so on [97–99]. National and local policy to influence such environments can greatly facilitate – or hinder – the adoption of healthy lifestyle patterns by individuals [100]. Recent research on health literacy has reframed the concept from a deficiency of the individual (redressed by ‘education’) to a deficiency of the system (redressed through community- and organisational-level changes to make services more understandable and accessible to everyone, whatever their cognitive capacity and system knowledge) [101, 102]. Such approaches illustrate how the axis of EBM can and should shift from evidence-based individual decisions (in which the evidence is generally simple, with a linear chain of causation and derived from randomised controlled trials) to evidence-based public health (in which evidence is complex, with non-linear chains of causation and derived from a wider range of research designs including natural experiments and community-based participatory research) [103, 104].

Similarly, healthcare organisations that were designed decades ago to deliver paternalistic care for single diseases will lack the structures, culture, systems, and routines needed to support a democratic, collaborative, and interdisciplinary approach to self-management in patients who increasingly have more than one chronic condition [105]. The research literature on experience based co-design suggests that designing services and treatments with patients, based on detailed analysis of the patient experience, is likely to produce organisations and systems that support evidence-based care [106].

## Summary

We have argued that the EBM paradigm is not as patient centred as it is sometimes assumed to be. We are concerned that the methods and approaches currently being adopted by the EBM community to ‘involve’ and ‘empower’ patients will not, in and of themselves, redress this deficiency.

The six ‘biases’ described in this review – the limited involvement of patients and carers in research; EBM’s hierarchical dismissal of personal experience and qualitative research more generally; its tendency to over-emphasise the use of decision tools at the expense of more humanistic elements of the consultation; its failure to recognise and address power imbalances; its implicit assumption that key decisions happen with a clinician in the room; and its neglect of the inverse care law – can all be traced back to the assumptions and preferred focus of the discipline of epidemiology: the science of experimental and observational studies of diseases in populations.

EBM’s epidemiological focus, which is appropriate and rigorous when considering populations or samples, places limited emphasis on aspects of healthcare that are key to the successful application of quantitative research evidence to the individual patient. The conceptual frames of EBM effectively configure the patient as an autonomous rational chooser, a model that does not readily translate into the everyday lives of real patients – multifaceted individuals with physical, cognitive, emotional, and social dimensions, who lead messy, idiosyncratic, networked, and often complicated lives in contexts that are shaped by cultural, economic, and political forces. As Mark Tonelli observed in 1999,

“[In evidence-based medicine], *the individuality of patients tends to be devalued, the focus of clinical practice is subtly shifted away from the care of individuals toward the care of populations, and the complex nature of sound clinical judgement is not fully appreciated.*”

However, whilst this problem has been described for decades, workable solutions have not arisen from within the EBM literature. In our view, this is because generating such solutions would require a fundamental change in perspective, an abandoning of certain deeply held principles and assumptions, and the introduction of new ideas and methodologies from disciplines beyond EBM. Given the policy push for greater patient and carer involvement in research, the time is surely ripe for those who adhere to the EBM paradigm to question its rigid ‘gold standard’ [107] and consider whether it is time to extend and enrich EBM’s evidence base.

In particular, EBM researchers should learn from the literature on civic engagement with a view to building a level of patient and public involvement in research that goes beyond the limited goal of increasing recruitment to research trials [108]. EBM practitioners should learn from the humanities (especially philosophy and literature) to ensure that individual (‘personally significant’) evidence, both subjective and objective, is given appropriate weight in clinical decision-making [28, 109]. They should take a more interdisciplinary and humanistic view of clinical consultations, drawing, for example, on the evidence from social psychology and medical education on the importance of the therapeutic relationship [38]. All this would require a greater focus on the deliberative analysis of real, unique individual cases rather than standardised fictional ones in teaching and professional development [110].

Those who seek to apply EBM in policy and practice should also consider the literature from social and political sciences and critical public health on power and inequality, especially research on power dynamics in healthcare encounters [10, 46], social determinants of health [100], and differential access to health services [89, 94]. Finally, those whose research focus is the patient should seek to engage with theories, methods, and empirical findings from (among other fields) digital sociology on self-monitoring, online peer support, and tacit knowledge exchange [7, 87].

In conclusion, as we have argued previously, EBM may not be ‘a movement in crisis’, but it is certainly at a crossroads [111]. The success of clinical epidemiology has taken the EBM movement to a stage where many of the unanswered research questions are no longer epidemiological but humanistic, social, and political. Nowhere is this truer than in EBM’s efforts to be patient-centred. We believe that the interdisciplinary approach described in this paper would allow EBM practitioners and policymakers to overcome or reduce what we have (perhaps somewhat provocatively) described as ‘biases’ against patients and carers.

### Details of contributors

We bring extensive collective experience as patients (medical and surgical, acute and long-term, physical and mental) and as parents and carers of people who are ill, dependent, or vulnerable. One of us (RS) used her experience of living with a long-term condition as the basis for a PhD in the sociology of healthcare [45]; another (SRy) has brought the perspective of a parent to social science research on chronic illness, disability, and clinical error [75]; another (TG) recently spent a year recovering from trauma [76].

## Abbreviations

DCCT: Diabetes Control and Complications Trial
EBM: Evidence-based medicine
